# Pharmacokinetic Simulation of Remifentanil Dosing at Different Effect-Site Targets During Target-Controlled Infusion for Cesarean Delivery

**DOI:** 10.3390/jcm15187054

**Published:** 2026-09-11

**Authors:** Ilja Osthoff, Monica Soare, Franz-Josef Vogl, JoEllen Welter, Alexander Dullenkopf

**Affiliations:** Spital Thurgau, 8501 Frauenfeld, Switzerland; ilja.osthoff@stgag.ch (I.O.); monica.soare@balgrist.ch (M.S.); franzjosef.vogl@stgag.ch (F.-J.V.); joellen.welter@stgag.ch (J.W.)

**Keywords:** cesarean delivery, general anesthesia, remifentanil, target controlled infusion, patient safety

## Abstract

**Background/Objectives**: Remifentanil may be useful during general anesthesia for cesarean delivery, although the amount administered before delivery using target-controlled infusion (TCI) at different effect-site targets has not been well quantified. This pharmacokinetic study investigated how much remifentanil would be delivered before birth at different initial effect-site targets. **Methods**: This retrospective simulation study used demographic data from 50 women undergoing cesarean delivery. Remifentanil TCI (Minto model) was simulated with initial effect-site targets of 1, 2, 4, and 6 ng/mL. One minute after the simulated plasma peak, the target was reduced to 1 ng/mL (t_0_). Doses before t_0_, duration of the infusion pause, and total dose before delivery were calculated. Simulated dosing patterns were summarized overall and stratified by elective and emergency procedures. **Results**: Infusion pauses after target reduction lasted 175, 346, and 503 s after initial targets of 2, 4, and 6 ng/mL, respectively. Delivery occurred during these pauses in 16%, 46%, and 72% of cases (19%, 71%, and 90% in emergency cases). Mean remifentanil dose at delivery was 33.7, 43.0, 70.0, and 101.0 μg for initial targets of 1, 2, 4, and 6 ng/mL, respectively. Following target reduction, the additional remifentanil administered before delivery decreased from a mean of 17.0 μg with an initial effect-site target of 1 ng/mL to 0.9 μg with an initial target of 6 ng/mL. **Conclusions**: This simulation demonstrated that only modest additional doses of remifentanil would be delivered to the mother between induction and delivery during target-controlled infusion for cesarean delivery, with the exact amount depending on the initial effect-site target concentration.

## 1. Introduction

Although neuraxial anesthesia is the preferred method for cesarean delivery, general anesthesia is required in certain situations [1,2,3]. These include emergency cases, contraindications to neuraxial techniques, patient refusal, or inadequate neuraxial block. General anesthesia for cesarean delivery is usually induced with intravenous hypnotic agents, and maintenance is typically achieved with inhalational agents [1,4,5,6]. Opioids are generally minimized or even avoided until delivery to prevent neonatal respiratory depression and other opioid related effects [1,2,7,8].

Remifentanil is a potent opioid with an ultra-short duration of action and it is used in various clinical settings, such as neurosurgery requiring rapid awakening, procedures with varying pain intensity, surgical settings with rapid transitions [9,10], as well as anesthesia for infants [11,12,13], and patient-controlled analgesia (PCA) during labor [14]. Additionally, remifentanil is advised during anesthesia induction in patients undergoing cesarean delivery who are suffering from pre-eclampsia [15]. While it is well suited for patients with liver or kidney disease since it is metabolized independently of liver and kidney function [16], there are potential complications such as severe respiratory suppression, muscle rigidity, hypotonia and bradycardia, especially with high doses [16]. Other issues are acute pain after discontinuation if not treated adequately otherwise, and potential hyperalgesia following prolonged or high-dose administration [17]. Given its unique pharmacokinetic properties, it has also been proposed for use during cesarean delivery [18,19].

An advanced method of remifentanil administration is target-controlled infusion (TCI) [20,21]. In TCI mode, a target effect-site (Ce) or plasma (Cp) concentration of remifentanil is selected (Cet or Cpt, respectively), and a computer-controlled infusion pump adjusts the administered dose according to a pharmacokinetic model incorporating patient sex, age, height and weight. To reach an effect-site target, the system delivers an initial dose that temporarily increases plasma concentration above the selected target. Following the plasma peak, the infusion rate decreases until plasma and effect-site concentrations reach equilibrium. When the target is subsequently reduced, the TCI system temporarily interrupts drug delivery, allowing plasma and effect-site concentrations to decline.

Remifentanil is infrequently administered before delivery, as the early infusion dosing profile and drug transfer to the newborn have not been adequately described. Quantifying the amount of remifentanil administered before birth may improve understanding of the theoretical framework of potential fetal drug exposure during target-controlled infusion. The dose delivered before birth across realistic cesarean delivery time intervals remains unknown. Simulation permits evaluation of these patterns without clinical opioid exposure. The aim of this bench simulation study was to determine how much remifentanil would be administered before birth using conventional target-controlled infusion with different initial effect-site targets during cesarean delivery.

## 2. Materials and Methods

This retrospective bench simulation study used demographic data from patients undergoing cesarean delivery as inputs for pharmacokinetic modeling. It was conducted in accordance with the Declaration of Helsinki. The local ethics committee (Ethics Committee East Switzerland; EKOS 24/026; 22 February 2024) determined that the project met the criteria for exemption from formal research ethics review. Use of patient data required documented general consent for secondary use of clinical data, which is routinely collected at the institution (general consent). The setting was a secondary Swiss cantonal hospital, performing approximately 250 cesarean deliveries annually.

### 2.1. Patient Data

Demographic and obstetric data were obtained from consecutive third trimester patients who underwent elective or emergency cesarean delivery at our hospital during 2024. Age, height, and weight at the time of surgery were extracted from the hospital clinical patient information system (KISIM; Cistec, Zurich, Switzerland). Retroactively from the date of ethical approval, patients who underwent cesarean delivery were screened for positive general consent and included up to a total of 50 patients.

### 2.2. Simulation of Remifentanil Target-Controlled Infusion

For each patient, a commercially available remifentanil TCI (Alaris Nexus PK Infusion Pump; BD, Alschwil, Switzerland) was simulated using the Minto pharmacokinetic model. The TCI system calculates individualized infusion rates using patient demographic characteristics (age, height, weight, and sex) according to the selected pharmacokinetic model. No modifications to the default model parameters were made. Simulations were performed for each patient using four initial effect-site target concentrations: 1, 2, 4, and 6 ng/mL. The remifentanil dose delivered up to the time of the plasma peak was recorded. The plasma peak was identified from the pump’s display during simulated drug administration.

It was assumed that one minute after the plasma peak (t0), the clinical situation would correspond to the typical early phase of general anesthesia (adequately anesthetized) for cesarean delivery, with induction completed, the trachea intubated, and the incision performed. This time point was selected based on institutional experience in non-pregnant emergency patients. At t0, the effect-site target concentration was reduced to 1 ng/mL, the initial maintenance target commonly used for general surgery at our institution. The duration of the infusion pause that occurs automatically after reducing the target concentration was recorded, along with the amount of remifentanil delivered every minute for ten minutes after t0. Infusion rates and cumulative remifentanil doses were recorded at one-minute intervals from the pump display.

### 2.3. Outcome Determination

The primary outcome was the total remifentanil dose administered to the mother before delivery for each initial effect-site target. The time of delivery (tD) was obtained from the anesthesia record of each patient. Using the recorded interval from incision to delivery (t0 to tD), the total remifentanil dose delivered from t0 to tD was calculated for each initial target concentration. For each target concentration, absolute and weight-adjusted (mcg/kg) remifentanil doses were determined. Outcomes were summarized for the entire cohort and stratified for elective and emergency procedures. The comparison between elective and emergency procedures was performed as an exploratory analysis and was not a predefined primary objective of the study.

### 2.4. Statistical Analysis

Descriptive statistics were used to summarize patient characteristics and simulated dosing data. No formal sample size calculation was performed. This exploratory simulation study used a convenience sample of 50 consecutive eligible patients with documented general consent. Continuous variables are presented as mean ± standard deviation, and categorical variables as absolute numbers and percentages. Comparisons between elective and emergency procedures were exploratory. Mean differences with 95% confidence intervals, calculated without assuming equal variances, were reported. Hypothesis testing was not performed, as the simulation outputs were deterministic for each set of patient inputs. All analyses were performed using Stata version 19 (StataCorp LLC, College Station, TX, USA).

## 3. Results

The study population comprised 50 patients with a mean age of 32.3 ± 4.3 years. Mean height and weight were 164.8 ± 5.9 cm and 82.8 ± 17.5 kg, respectively, corresponding to a BMI of 30.5 ± 6.6 kg/m^2^. Of these, 29 patients (58%) underwent elective cesarean delivery. Mean time from incision to delivery was 6.1 ± 3.2 min overall, 7.2 ± 3.3 min in elective cases, and 4.7 ± 2.6 min in emergency cases.

The total simulated remifentanil dose at t_0_ (one minute after the peak plasma concentration) increased proportionally with the effect-site concentration target. Mean doses were 16.7 ± 1.0 mcg (0.2 ± 0.0 mcg/kg) for a target of 1 ng/mL, 33.4 ± 2.0 mcg (0.4 ± 0.0 mcg/kg) for 2 ng/mL, 66.8 ± 4.1 mcg (0.8 ± 0.1 mcg/kg) for 4 ng/mL, and 100.1 ± 6.0 mcg (1.2 ± 0.1 mcg/kg) for 6 ng/mL.

After the effect-site target was reduced at t_0_ to the anesthesia maintenance level of 1 ng/mL, the infusion pump automatically paused remifentanil administration for 174.6 ± 8.8 s, 345.5 ± 14.2 s, and 503.1 ± 19.4 s following initial targets of 2, 4, and 6 ng/mL, respectively. When the initial target was 1 ng/mL, no deliveries would have occurred during the pause period. Deliveries would have occurred during the infusion pause in 8 cases (16%) overall when the initial target was 2 ng/mL, in 23 cases (46%) at 4 ng/mL, and in 36 cases (72%) at 6 ng/mL. Among emergency cases, the corresponding proportions were 19%, 71%, and 90%, respectively.

For the primary outcome, the total remifentanil dose at delivery was 33.7 ± 10.4 mcg (0.4 ± 0.1 mcg/kg) when induction effect-site target was set to 1 ng/mL, and 43.0 ± 8.7 mcg (0.5 ± 0.1 mcg/kg), 70.0 ± 6.0 mcg (0.9 ± 0.1 mcg/kg), and 101.0 ± 6.4 mcg (1.3 ± 0.2 mcg/kg) when set to 2, 4, and 6 ng/mL, respectively. Remifentanil doses administered from induction until delivery, stratified by elective and emergency cases, are summarized in Table 1.

Compared with elective procedures, mean remifentanil doses at delivery were lower in emergency procedures by 8.0 μg (95% CI, 2.6 to 13.5), 7.5 μg (95% CI, 3.2 to 11.9), 4.1 μg (95% CI, 1.1 to 7.2), and 2.4 μg (95% CI, −1.2 to 5.9) at an initial targets of 1, 2, 4, and 6 ng/mL, respectively.

## 4. Discussion

In this pharmacokinetic simulation study, higher initial effect-site target concentrations of remifentanil produced proportionally higher total doses at delivery and longer pauses in infusion after the target was reduced. Delivery would have occurred during these pauses in a greater proportion of emergency than elective cases. Overall, only moderate amounts of remifentanil would have been administered to the mother before delivery across all simulated scenarios.

Regional anesthesia remains the preferred technique for cesarean delivery [3,19,22,23]. It allows the mother to be awake at birth, avoids airway management in pregnant patients, and reduces fetal exposure to anesthetic drugs. However, when general anesthesia is required, clear guidance on optimal anesthetic management remains limited, including which medications should be used [1,24]. General anesthesia for cesarean delivery is traditionally induced with intravenous agents such as propofol, thiopental, supplemented, if necessary, for example with ketamine, followed by maintenance with inhalational agents [4,25,26].

Opioids are commonly used in anesthesia not only to alleviate pain but to reduce stress responses. Remifentanil is an ultra-short acting opioid structurally related to fentanyl, but because it is metabolized predominantly by non-specific extrahepatic esterases, it has a high plasma clearance of 3 L/min and a terminal half-life of 10–21 min [9,10]—almost independent of exposure time. These properties make remifentanil particularly suitable for reducing residual opioid effects after sedation or general anesthesia. As a result, remifentanil is incorporated into many anesthetic regimens, including pediatric surgery or non-neuraxial labor analgesia [12,14]. Consequently, many anesthetists are familiar with its use across a wide range of clinical settings. Remifentanil has not been extensively studied in obstetric patients. It crosses the placenta but appears to be rapidly metabolized, redistributed, or both. Mean and SD ratios of umbilical vein: maternal artery and umbilical artery: umbilical vein have been reported at 0.88 ± 0.78 and 0.29 ± 0.07, respectively [27]. Maternal sedation and respiratory changes occur, but with limited adverse neonatal or maternal effects.

Target-controlled infusion requires an initial bolus to achieve the selected effect-site concentration, followed by a continuous infusion to maintain the target [13,28]. In general anesthesia, the target is typically reduced after anesthesia induction. When the target is lowered, the TCI system achieves this by temporarily stopping the infusion. In the context of cesarean delivery, this pause may coincide with the time before birth, meaning that no further remifentanil would be delivered beyond the initial bolus. It is important to recognize that the pharmacokinetic/pharmacodynamic studies used to develop current TCI models did not include pregnant patients, and when TCI is used in clinical practice, careful monitoring of anesthetic depth is strongly recommended [28,29].

For routine surgery at our institution, remifentanil TCI is typically initiated with an induction effect-site target of 2 to 4 ng/mL, combined with propofol TCI and monitored using bispectral index EEG (BIS). The effect-site target is usually reduced approximately one minute after the plasma peak is reached, and the same approach was applied in this bench simulation. With an initial target of 2 ng/mL, the simulated induction bolus of remifentanil averaged 33.4 mcg, and 66.8 mcg for a target of 4 mcg/mL. The additional amount that would have been administered to the mother up to delivery after an initial target of 2 ng/mL was small, averaging only 9.6 mcg in the overall cohort and 5.4 mcg in emergency cases. These doses should be considered in relation to the concentrations of inhalational anesthetics required to maintain anesthesia, as both remifentanil and volatile agents readily cross the placenta and may influence the neonatal status. After an initial Cet of 4 ng/mL, no additional remifentanil would have been administered to the mother between induction and delivery in 46% of all cases and 71% of emergency cases.

This study has several limitations that should be considered when interpreting the findings. First, this study relied on pharmacokinetic simulations rather than administration of remifentanil to patients. Consequently, simulated dosing reflected the behavior of the Minto pharmacokinetic model and should not be interpreted as independent validation of relationships that are inherent to that model. Furthermore, the Minto model was developed using data from non-pregnant adults and has not been specifically validated for pregnant patients, whose physiological changes may alter remifentanil pharmacokinetics [30]. Beyond that, emergency cesarean delivery represents conditions such as fetal distress, placental abruption, uterine rupture, severe preeclampsia, cord prolapse, etc. These scenarios differ considerably with respect to maternal physiology, fetal condition, and placental perfusion, which limits potential interpretation of findings and deserves further study. In addition, the model estimates lean body mass using the James equation, which has recognized limitations in patients with obesity and may affect dosing predictions in this subgroup. Second, the results only describe the amount of remifentanil that the infusion pump would administer to the mother and do not demonstrate placental transfer kinetics, fetal drug exposure, fetal pharmacokinetics, or neonatal pharmacodynamic effects. Once remifentanil crosses the placenta, fetal drug disposition depends on its own pharmacokinetic characteristics. Consequently, interruption of maternal infusion should not be interpreted as evidence that fetal plasma concentrations decrease in a similar fashion. Third, the simulations assumed standardized clinical conditions at t_0_, although induction technique, airway management, and surgical workflow vary in practice. T_0_, however, was chosen in our study from institutional experience in emergency patients who were not pregnant. Different timings for target reduction would influence the simulated results. Fourth, preparation and programming of a TCI system in emergency settings may require more time than initiation of inhalational anesthesia, although remifentanil and propofol are increasingly available and TCI use is becoming more common. Fifth, incision-to-delivery times were taken from clinical records but applied uniformly to all simulated target concentrations. Consequently, the model did not account for any influence of remifentanil dosing on obstetric or anesthetic management. Sixth, the sample size reflected the available patient population rather than a predefined power calculation, and the study was conducted at a single center, which may limit generalizability. Finally, neonatal exposure was not simulated, and no conclusions can be drawn regarding neonatal safety. Future clinical studies should evaluate whether TCI-guided remifentanil administration during cesarean delivery improves maternal anesthetic conditions while maintaining acceptable neonatal outcomes.

## 5. Conclusions

In conclusion, simulating target-controlled remifentanil administration for cesarean delivery demonstrated that, when using typical clinical settings with an initial higher effect-site target followed by a reduction to a maintenance level, only limited additional amounts of remifentanil would be delivered to the mother between induction and delivery. Since these findings are based on pharmacokinetic simulation and do not demonstrate clinical reassurance, prospective clinical studies are needed to evaluate maternal anesthetic conditions and especially neonatal outcomes.

## Figures and Tables

**Table 1:** Simulated remifentanil dose administered at delivery (tD), and additional remifentanil administered after the initial dose to delivery (t0 to tD), with effect-site concentration initially selected as 1, 2, 4, and 6 ng/mL, respectively, and reduction at t0 to maintenance effect-site concentration of 1 ng/mL. Overall data, as well as data for elective and emergency cases (*n* = 50). Data are mean ± standard deviation.

Remifentanil Dose at	t_D_			t_0_ to t_D_		
	Cohort	Elective Surgery	Emergency Surgery	Cohort	Elective Surgery	Emergency Surgery
**Effect-site concentration initially: 1 ng/mL**						
**mcg**	33.7 ± 10.4	37.0 ± 10.6	29.0 ± 8.4	17.0 ± 10.0	20.3 ± 10.2	12.4 ± 8.3
**mcg/kg**	0.4 ± 0.1	0.5 ± 0.1	0.4 ± 0.12	0.2 ± 0.1	0.2 ± 0.1	0.2 ± 0.1
**Effect-site concentration initially: 2 ng/mL**						
**mcg**	43.0 ± 8.7	46.1 ± 9.0	38.6 ± 6.1	9.6 ± 8.1	12.6 ± 8.2	5.4 ± 6.1
**mcg/kg**	0.5 ± 0.1	0.6 ± 0.1	0.5 ± 0.1	0.1 ± 0.1	0.2 ± 0.1	0.1 ± 0.1
**Effect-site concentration initially: 4 ng/mL**						
**mcg**	70.0 ± 6.0	71.8 ± 6.6	67.6 ± 4.2	3.3 ± 4.2	4.7 ± 4.7	1.3 ± 2.5
**mcg/kg**	0.9 ± 0.1	0.9 ± 0.1	0.9 ± 0.1	0.0 ± 0.1	0.1 ± 0.1	0.0 ± 0.0
**Effect-site concentration initially: 6 ng/mL**						
**mcg**	101.0 ± 6.4	102.0 ± 7.0	99.6 ± 5.5	0.9 ± 2.0	1.3 ± 2.4	0.3 ± 0.7
**mcg/kg**	1.3 ± 0.2	1.3 ± 0.2	1.3 ± 0.1	0.0 ± 0.0	0.0 ± 0.0	0.0 ± 0.0

## Data Availability

The datasets used and/or analyzed during the current study are available from the corresponding author on reasonable request.

## Abbreviations

The following abbreviations are used in this manuscript:

Ce	Effect-site concentration
Cp	Plasma concentration
PCA	Patient controlled analgesia
TCI	Target controlled infusion
